# BASIS: High-performance bioinformatics platform for processing of large-scale mass spectrometry imaging data in chemically augmented histology

**DOI:** 10.1038/s41598-018-22499-z

**Published:** 2018-03-06

**Authors:** Kirill Veselkov, Jonathan Sleeman, Emmanuelle Claude, Johannes P. C. Vissers, Dieter Galea, Anna Mroz, Ivan Laponogov, Mark Towers, Robert Tonge, Reza Mirnezami, Zoltan Takats, Jeremy K. Nicholson, James I. Langridge

**Affiliations:** 1 Computational and Systems Medicine, Department of Surgery and Cancer, Imperial College London, London, UK; 2 University of Heidelberg, Medical Faculty Mannheim, Center for Biomedicine and Medical Technology, Mannheim, Germany; 3 KIT Karlsruhe, Institute of Toxicology and Genetics, Karlsruhe, Germany; 4 Waters Corporation, Wilmslow, UK

## Abstract

Mass Spectrometry Imaging (MSI) holds significant promise in augmenting digital histopathologic analysis by generating highly robust big data about the metabolic, lipidomic and proteomic molecular content of the samples. In the process, a vast quantity of unrefined data, that can amount to several hundred gigabytes per tissue section, is produced. Managing, analysing and interpreting this data is a significant challenge and represents a major barrier to the translational application of MSI. Existing data analysis solutions for MSI rely on a set of heterogeneous bioinformatics packages that are not scalable for the reproducible processing of large-scale (hundreds to thousands) biological sample sets. Here, we present a computational platform (pyBASIS) capable of optimized and scalable processing of MSI data for improved information recovery and comparative analysis across tissue specimens using machine learning and related pattern recognition approaches. The proposed solution also provides a means of seamlessly integrating experimental laboratory data with downstream bioinformatics interpretation/analyses, resulting in a truly integrated system for translational MSI.

## Introduction

Cancer costs the European Union (EU) an estimated 124 billion euros every year, with an annual incidence of 3.45 million cases and 1.75 million associated deaths^1^. These grim realities call for urgent improvements in our fundamental understanding of cancer biology, with which to enhance current, and future, strategies for prevention, early diagnosis, and personalised therapy^2^. While current high-throughput (high-molecular content) analytical techniques have the potential to deliver a transformative change in deep cancer phenotyping, the rate-limiting step is the challenge of managing, analysing and interpreting the vast molecular datasets that these technologies generate^3,4^. Without progress and tailored strategies in the computational interpretation of these highly complex datasets, it is unlikely that these technologies will be able to realize their significant translational potential^5,6^.

Mass Spectrometry Imaging (MSI) is an emerging technology in research pathology that generates gigabytes of raw mass spectrometry data of potential biological and clinical importance^7,8^. The current need for MSI-based bioinformatics solutions can be summarized as: (*i*) large amount of data generated even for a single tissue due to the non-targeted nature of MSI; (*ii*) high demand for companion prognostic and diagnostic markers for the stratification of cancer therapy with high throughput and better affordability relatively to other techniques, which can be provided by MS; and (*iii*) the overall need for machine-learning-driven automation and standardisation of image molecular assessments to mitigate the current lack of well-qualified histopathologists for manual data interpretation^9^.

**Figure 1:**
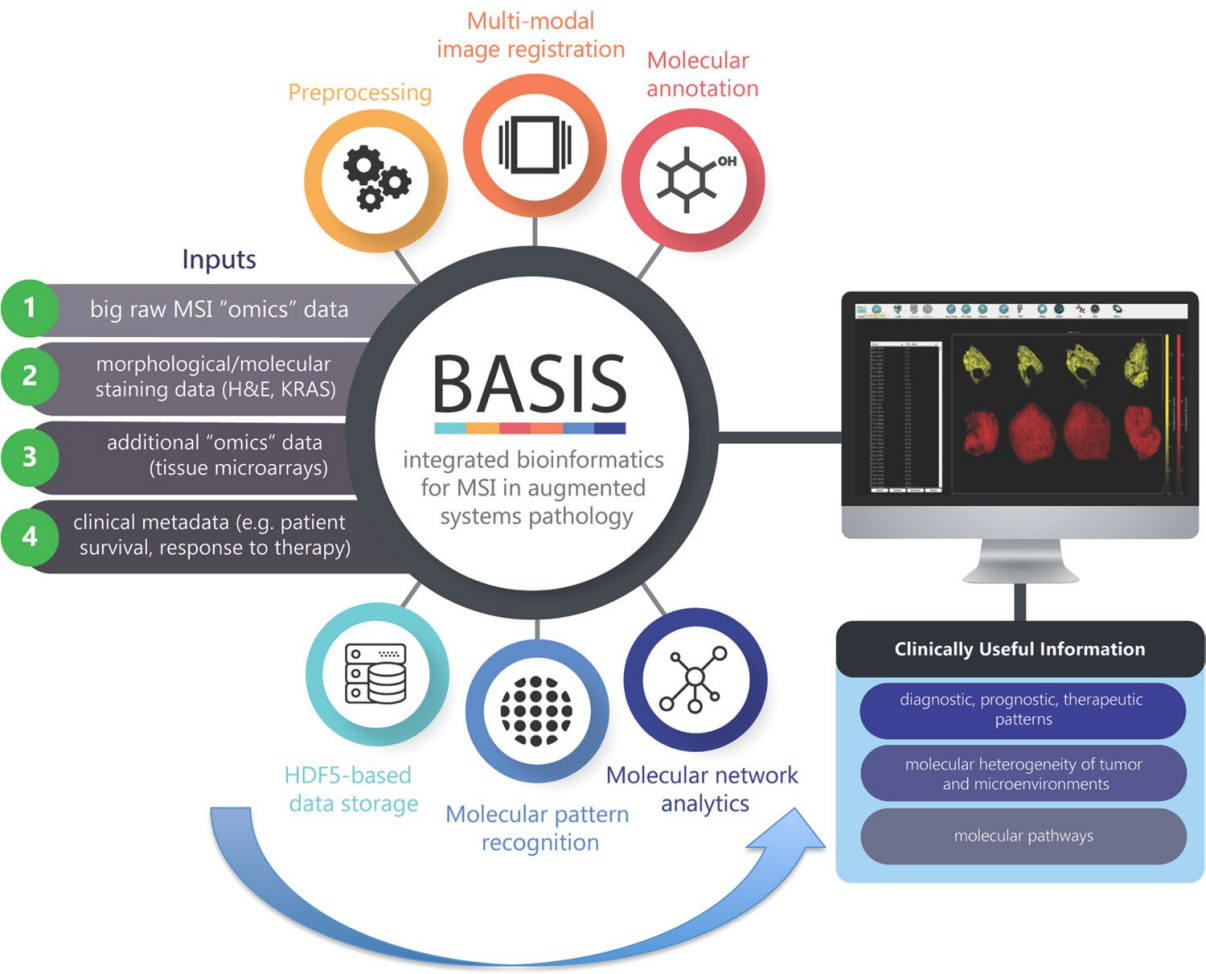
The translational data analytics pipeline for large-scale MS imaging data in clinical research settings. Incorporation of large scale MSI-derived data into conventional patient phenotyping approaches will require upstream handling and assimilation of multi-source, heterogeneous inputs and subsequent downstream generation of clinically relevant biological information. Linking these two steps requires a reproducible and robust bioinformatics pipeline that can seamlessly pre-process and analyse large scale MSI datasets. A fundamental facet of this pipeline will be its transparency and computational consistency – all pre-processed workflows and related meta-data will be registered and stored in open access. Here we introduce the pyBASIS (Bioinformatics for mAss Spectrometry Imaging in augmented Systems pathology) computational package that aims to address these requirements. The module icons displayed in this diagram were obtained from Flaticon under a free-license.

Initial studies using MSI were first described using secondary ion mass spectrometry (SIMS), but the technique has been more widely adopted since the utilisation of Matrix-Assisted Laser Desorption Ionization (MALDI)^10^ and, more recently, ambient ionisation techniques, in particular Desorption ElectroSpray Ionisation (DESI) mass spectrometry^11^. While recent advances in MSI technologies provide a wealth of specific molecular information for diverse applications such as histopathology and precision medicine, the major impediment to progress currently centres on the lack of a complete analytical solution based on chemo-informatic strategies^10,11^. In broad terms, these strategies need to include a series of designated steps covering: (*i*) raw analytical signal pre-processing to distinguish signal from noise and to reduce bio-analytical complexities of MSI data; (*ii*) imaging informatics for co-registration, correlation and integration of MSI data and images with medical imaging and histopathological/immune-histochemical data; (*iii*) putative molecular ion identification and annotation; (*iv*) machine learning approaches for the amalgamation of MSI with other diagnostics and prognostics techniques; and (*v*) network-driven analysis for pathway-oriented tissue analytics^9,12,13^. If carried out appropriately, these strategies can be expected to efficiently transform a large volume of MSI data into more concise biologically and clinically useful information (Fig. 1).

An optimised and reproducible workflow for pre-processing of MSI data represents the fundamental requirement for enhancing pattern recognition and information recovery across multiple studies^9,12,13^. A typical pre-processing workflow starts with the reduction of data volume by means of peak/feature detection. This step is commonly performed during MSI data acquisition as it is dependent upon the physical properties of the employed analytical platform^14,15^. The generated peak intensity matrix requires correction of the measured mass to charge (m/z) values of molecular ion species. To improve precision of mass determination, mass spectrometers are typically calibrated prior to an experiment by comparing the acquired and theoretical mass spectra of known “reference” compounds to characterise the mass scale. This procedure, referred to as “external calibration”, is commonly used to calibrate mass spectrometers. Subsequent to this base calibration, a variety of experimental/environmental factors, such as temperature and voltage variation on the orthogonal acceleration (oa)-TOF m/z scale, impact the accuracy of molecular m/z measurements. This is especially the case for large-scale MSI data, which are frequently acquired over lengthy, often discontinuous, time frames. Most currently used approaches for mass correction rely on a set of molecular ion species with known m/z values assumed to be ubiquitously distributed within tissue sections. However, these reference values are not always available for untargeted molecular phenotyping studies^16^. This is then followed by a strategy for intra- and inter-sample data normalisation to account for variations in overall spectral intensity that may be seen as a consequence of factors (such as inhomogeneous matrix deposition), variable tissue section thickness, mass spectrometry variations (such as detector gain effects) or inconsistent sample preparation. Conventional normalisation strategies often involve scaling of spectral intensities according to total area (or mean intensity); however, their performance can be easily compromised by the presence of a few peaks of large and variable intensity^17,18^. An additional problem inherent to MS-based analysis of complex biological mixtures is the fact that molecules present in greater intensities within a given sample will tend to exhibit larger variations when subjected to repeated measurement. This asymmetric variation across the measurement range, known in statistical terms as heteroscedasticity, represents a significant barrier to the effective application of commonly used multivariate techniques for the downstream statistical interrogation of MSI datasets. To date, a number of different strategies have been proposed in the literature to stabilize variance, and we have recently validated several such techniques in the context of DESI and MALDI-MSI^9^. Of these, the started log-transformation has been shown to be the preferred option. Finally, most current MSI data treatment algorithms include a step designed to remove solvent/matrix related spectral signals. This usually involves comparative analysis between on-tissue and off-tissue spectral intensity, or assessment of spatial “chaos”/randomness of molecular ion channels of individual specimens^9,16,19^. However, distinguishing tissue from background can be challenged by the presence of these very signals. This type of workflow can be applied to small-scale studies where these shortcomings can be addressed manually, but the application of these approaches to large scale MSI datasets is unfeasible due to the impracticality of manually assessing individual sample data.

Currently used data analysis tools for MSI data are integrated into open-source software packages such as: MSIReader^20^, Cardinal^16^, OmniSpec^21^, or freely available openMSI platforms^22,23^, BioMap (Novartis), SpectViewer (CEA), DataCubeExplorer (AMOLF), Mirion (JLU)^24^, or within commercial packages from instrument manufacturers such as: Xcalibur/ImageQuest (Thermo Fisher Scientific), High Definition Imaging (HDI, Waters Corporation) and SCiLS (Bruker Daltonics). These packages are limited to basic pre-processing and pattern recognition analysis of individual samples^9,15^. The SpectralAnalysis platform has advanced solutions for multiple sample analysis but lacks critical pre-processing capability (such as variance stabilizing normalization and robust solvent/matrix filtering) and relies on the relatively inefficient “imzML” file format for data storage and read/write speed^15^. The imzML equivalent data formats for chromatography-mass spectrometry (mzML or mzXML) have been previously evaluated to be 3–4 times slower in read and write speeds, and more than 50% larger in space compared to HDF5-based mz5 data format^25^. For this reason, large-scale MSI data set analyses have not been forthcoming and are hindering the wider adoption of the technology for clinical research.

Here, we introduce the python BASIS (pyBASIS) platform, which has been designed for reproducible and tailored processing of large scale MSI datasets of hundreds of tissue specimens. Key features in the design of pyBASIS include (a) high performance data file (HDF5) streaming architecture; (b) iterative data processing (one sample at a time); and (c) robust pre-processing pipelines scalable to hundreds, and potentially thousands, of tissue specimens. We have implemented an open-source imzML data import that enables the use of MSI data from different systems^26,27^. The proposed solution also provides a means of seamlessly integrating experimental laboratory data with downstream bioinformatics interpretation/analyses, *via* the Symphony^TM^ platform, resulting in a truly integrated system for translational MSI. Here, we demonstrate the application of this workflow on MSI (MALDI and DESI) datasets acquired from animal as well as human cancer studies. However, the BASIS platform can be applied across a wide range of large-scale MSI data studies

## Results and Discussion

The relative inability to effectively interrogate large-scale (hundreds to potentially thousands of samples) datasets represents a major roadblock in translational MSI. Key requirements for large scale MSI studies are analytical transparency, reproducibility and replicability, workflow versatility, and scalability. Of the currently available solutions, only SpectralAnalysis^15^ has been designed for the analysis of multiple datasets. However, this platform lacks critical pre-processing capability and utilises an inefficient imzML file format, which despite being designed as an inter-operable universal file format, is cumbersome in terms of read/write speed as well as data storage and accessibility. The pyBASIS platform presented here has been designed to address these limitations.

**Figure 2:**
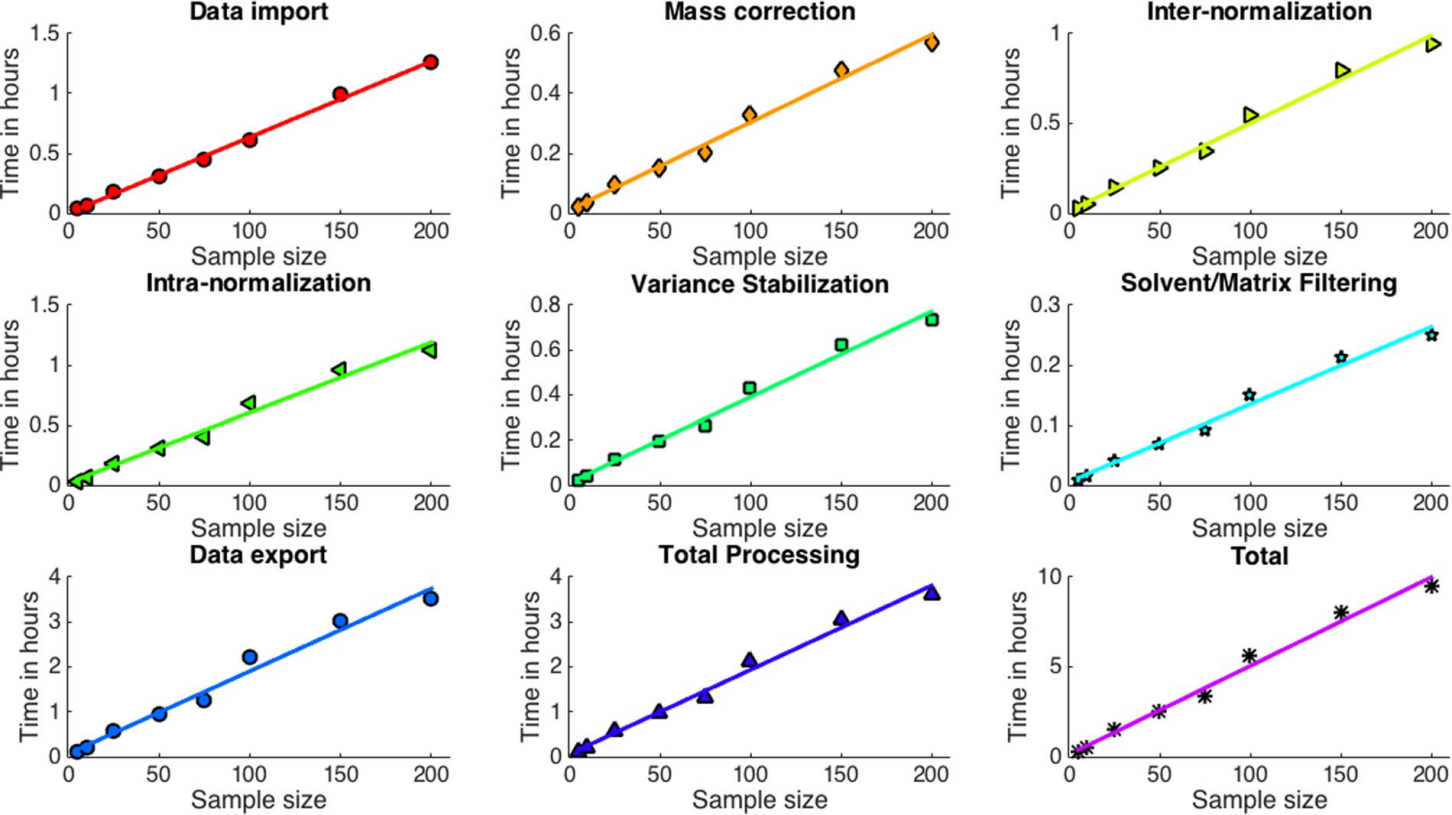
Linear O(N) performance of each developed pipeline module. The dependency between the number of processed MALDI/DESI-MSI sample datasets acquired on SYNAPT G2-S*i* platforms MSI sample datasets and the processing time (0.5 GB of peak picked (~2000 peaks) data per sample; ~100 GB of data for 200 samples). All processing was done using a single core/thread of a standalone workstation PC (8 core Intel® Xeon ® E5-2630 v3 @2.4 GHz, 64 Gb RAM, 3 Tb HDD). The total processing time excluded data import and export, which are included in the Total plot.
(C) Workflow reproducibility and replicability – all workflow steps and generated pre-processing metadata (e.g. common m/z feature vector, choice of normalisation strategy, user defined parameter settings) are stored as part of the database file. This feature allows consistent and reliable comparison between newly collected and archived data.

The unique features of the pyBASIS platform are:

(A) A modular, customisable and readily extendable workflow with enhanced data pre-processing capabilities, covering all prerequisite steps for MSI data treatment. Each module comprises a series of analytical methods designed to deal with a given pre-processing task, and these methods can be extended/modified by the user as required.
(B) A scalable “out-of-core” data pre-processing pipeline. Out-of-core (or “external memory”) processing is a technique used to process data that is too exhaustive to fit in a computer’s main memory (RAM). All pyBASIS algorithms have been designed to operate iteratively for enhanced scalability by using high performance HDF5 technologies. Currently available solutions largely lack the capability to perform iterative analysis as they have been primarily developed to permit manual interrogation of single samples or small-scale projects. A design feature incorporated into pyBASIS allows individual sample data to be uploaded one at a time into the specific module. Data are then processed, deposited back into the data repository, and deleted from memory, with this procedure being repeated iteratively. Figure 2 illustrates the linear O(N) dependency between the number of samples processed and the processing time, taking about 4 hours for processing 200 MSI dataset samples. Since only one sample is stored in memory at any given time, the workflow memory load is constant. The algorithms’ performance will remain linear with the sample size increase. Any potential slow-down can be due to the reading and writing of data from the HDF5 file as the database file size increases. However, this slowdown has been shown not to occur to at least tens of terabytes of data^28^, *i.e*. thousands of samples in the above example.

### Mass Correction

Taking as input peak intensity data matrices, the pyBASIS pipeline performs correction of inherent inaccuracies in mass measurements, which is an essential prerequisite for downstream comparative analysis and annotation of large-scale MSI data. This is especially the case for large-scale MSI data, which are frequently acquired over lengthy, often discontinuous, time-frames. Most currently used approaches for mass correction rely on a set of molecular ion species with known m/z values assumed to be ubiquitously distributed within tissue sections^15,16^. This assumption is commonly fulfilled for small-scale studies, but not necessarily in larger scale studies, where greater molecular compositional variation is expected between samples. Therefore, pyBASIS employs a unique kernel-based “clustering” approach to group and align chemically-related ionic species to a common m/z vector. The advantage of this approach for untargeted molecular phenotyping is that it does not require any prior knowledge of internal or reference peaks with known m/z values for mass drift correction. For cases where a user provides one or more “reference” ionic species with known m/z values, an “internal lock” mass correction algorithm has also been implemented^16^. Using this procedure, the m/z drift for each sample data-set is calculated by taking the median of the mass shifts across all matched pairs of experimentally observed and theoretically calculated m/z ratios, within the mass drift window.

### Intra-sample normalization

Systematic differences in the total amount of desorption and ionization of molecular ions within and between sample datasets are frequently observed in MALDI and DESI-MSI high-throughput studies. The major factors responsible for these differences include inhomogeneity of external matrix deposition, variability of tissue thickness, and variation in ionization and detector efficiencies^9,29^. A total ion current normalisation approach has typically been applied to compensate for these effects^15,29^. However, this normalisation method can be easily compromised by the presence of a few large and variable intensity peaks. If it is assumed that there is a subset of molecular ion species that do not exhibit changes between tissue spectra, then the ratio of these non-differentially abundant metabolites should approximately equate to one. We have previously shown that this ratio of stable peaks can be robustly derived by calculating the median of molecular ion ratios across the entire spectrum with respect to the reference one, typically calculated as a median spectrum^9,18^. The performance of this method is robust against at least 50% peak intensities exhibiting asymmetrical increase or decrease in response to biological factors such as tissue morphological variation. Figure S1 illustrates the concept of intra-sample normalization. The changes in overall intensity can be diagnosed with the use of box-plots of log-fold changes of individual spectra. In the absence of overall intensity drift, these log-fold changes should be centered around zero.

### Inter-sample normalization

**Figure 3:**
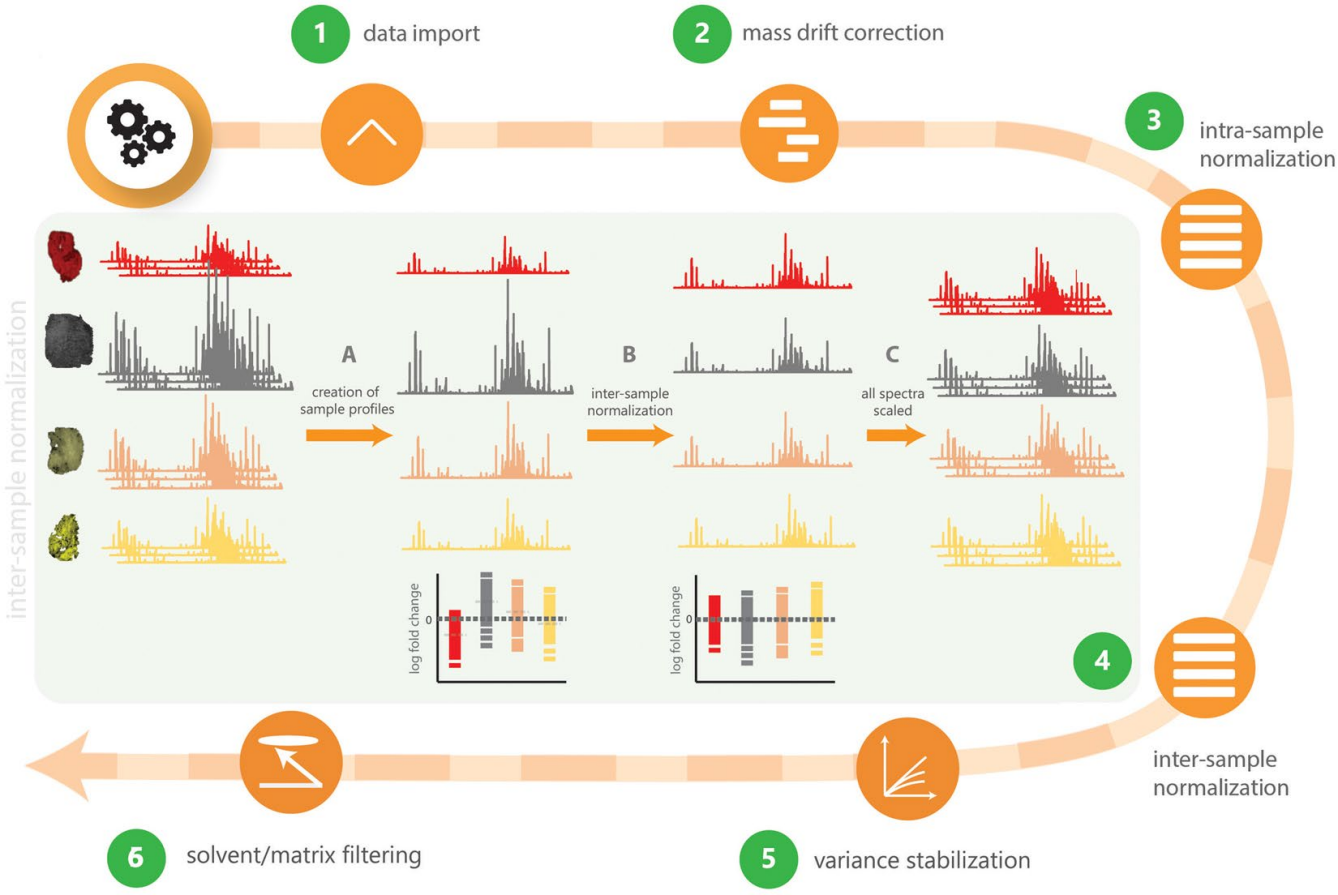
Integrated bioinformatics pipeline (pyBASIS) operating within a Symphony^TM^ environment for optimised processing and analysis of large scale MSI datasets. Inter-sample normalisation functionality is illustrated with creation of individual sample profiles (**A**), followed by derivation of sample-specific normalisation factors (**B**) and scaling of all spectral intensities using derived normalisation factors (**C**). Individual steps for the pipeline (1–6) are described in detail in the main text.

Variations in instrument parameter settings, environmental conditions and sample preparation protocols will lead to non-biologically relevant variability in overall peak intensity in acquired datasets. In order to ensure comparability in overall intensity within a batch and between multiple sets of samples, a variety of methods can be used to estimate normalization factors (see inter-sample normalization strategies in the methods section). The main difference of inter-sample normalization compared to intra-sample normalization is that the scaling factor is applied uniformly for all spectra of a given sample. The concept of inter-sample normalization is exemplified in Fig. 3. As with intra-sample normalization, the total ion current method can be easily compromised by a few large peaks^9,18^. The median fold change approach described above offers a robust way to estimate the overall intensity difference for a given sample when the changes in metabolite composition are expected. The difference to intra-sample normalization is that the median fold changes are calculated based on homogenised (‘average’) tissue profiles. Similarly, the box plots of log-fold changes of tissue profiles can be used for diagnostics to assess the normalization performance.

### Variance-stabilizing transformation

An additional problem inherent to MS-based analysis of complex biological mixtures is the fact that molecules present in greater intensities within a given sample will tend to exhibit larger variations when subjected to repeated measurement. As a result of this technical variance, metabolites with higher peak intensities would exhibit larger variability when repeatedly measured, and thus weak signals can be buried in the noise of strong signals^18,30^. The objective of the variance stabilizing transformation is to ensure that technical variance remains approximately the same, irrespective of signal intensity^29^.

**Figure 4:**
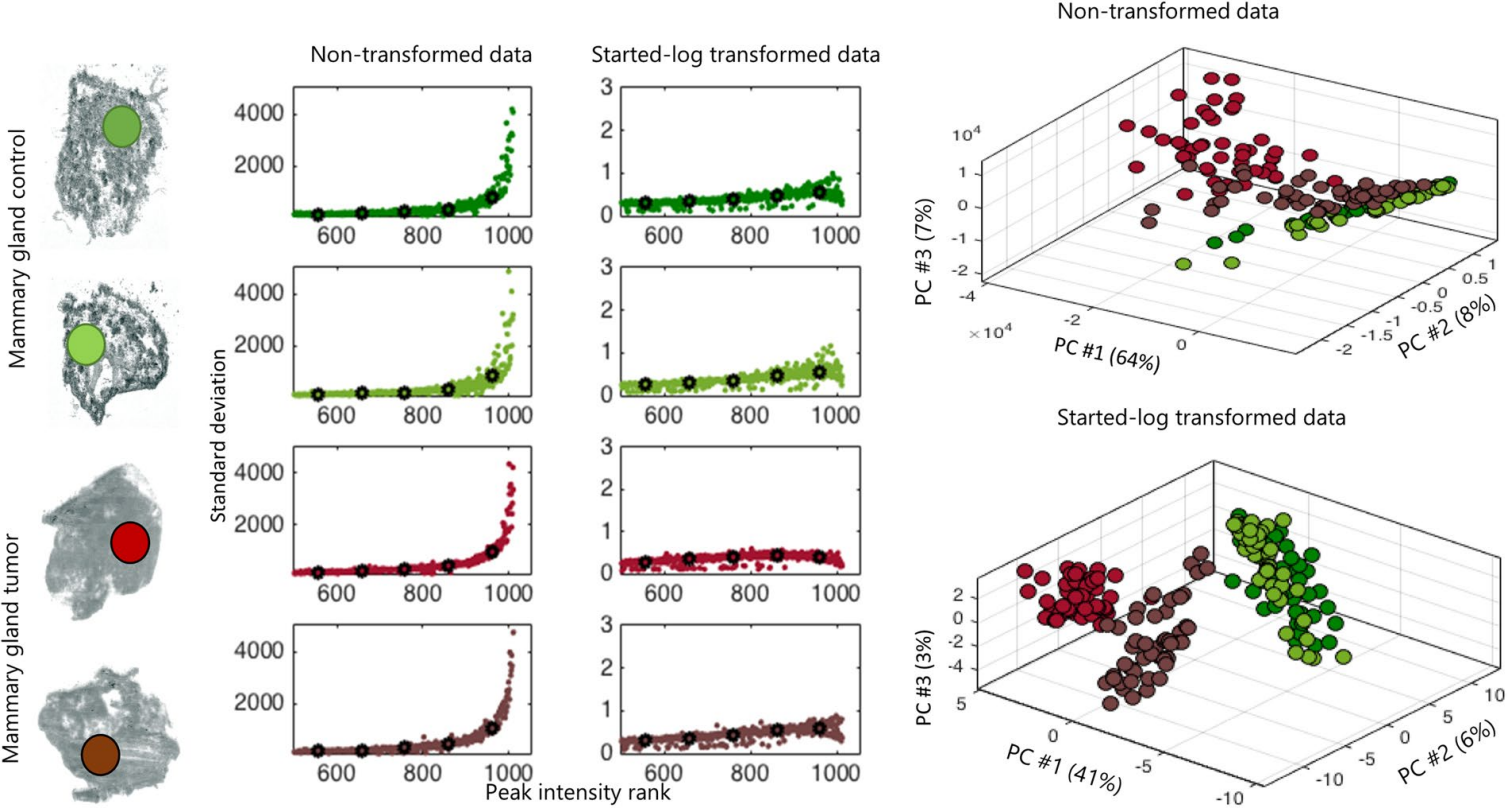
Impact of variance-stabilizing transformation on information recovery *via* unsupervised PCA-based analysis.

To assess the performance of the log-based strategy we selected MALDI imaging data from four different tissue sections, determined by a pathologist to be of the same morphological tissue type (i.e. where substantial biological variation is not expected within a given region). Figure 4 illustrates the standard deviation as a function of the ranked mean peak intensity within homogenous tissue regions. In the absence of heteroscedastic noise structure, the running median of the standard deviation should verge on horizontal, with minor oscillations only but no significant positive or negative deflection^30^. In non-transformed data this condition is not met and the variation of peak intensity is seen to increase with the rank of mean intensity (i.e. as intensity increases). This asymmetric variation across the measurement range represents a significant barrier to the effective application of commonly used multivariate techniques for the downstream statistical interrogation of MSI datasets. This is exemplified in the PCA plots where before applying the log-based transformation, the resulting principal component (PC) scores are heavily affected by the random variation of high-intensity molecular ion peaks and therefore are poorly representative of the overall variation structure within the dataset which would be expected to be primarily due to tissue morphology. Following started log-based transformation, improved stability of transformed peak intensities is observed across the measurement intensity range. This ensures that the data structure is consistent with the assumption of downstream pattern recognition techniques.

### Solvent/matrix filtering

DESI and MALDI MSI datasets from tissues contain signals comprising ionic species of biological molecules as well as those originating from solvent- or matrix-related adducts or fragments. The biologically irrelevant signal induced by matrix/solvent related peaks needs to be filtered out for improved information recovery by downstream statistical analyses. Two main approaches have been proposed in this regard^9,19^. One approach compares the mean peak intensity of tissue related pixels with background^9,16^. This approach is limited because defining tissue related pixels can be compromised by the very presence of these matrix/solvent related peaks. The other approach uses the randomness/degree of molecular chaos as a surrogate measure to distinguish biological from non-biological molecular ion features. The problem with this approach is that setting a threshold for randomness is somewhat arbitrary and computationally exhaustive when applied to large scale studies^19^.

In computational terms, the spatial distribution of background molecular ion intensities will be negatively correlated with the spatial distribution of tissue-related m/z species. Taking advantage of this property, the cluster-driven peak filtering strategy employed in pyBASIS calculates, in iterative fashion, the correlation matrix of the similarity/difference of signal distribution between m/z features across datasets. Any clustering algorithm can be applied to this correlation matrix to distinguish two “clusters” of m/z species of solvent/matrix and tissue-related origin, respectively. The k-means and Gaussian mixture model algorithms are used in the pyBASIS package. Because of its iterative nature, this robust solvent/matrix peak filtering strategy is the only strategy that can be applied to large-scale datasets. Figure S2 illustrates our cluster-driven matrix/solvent peak filtering strategy. In general, the variability within tissue-related features, including delocalized ones, is expected to be smaller than when compared to the variability of the background. Therefore, any delocalized tissue features are still expected to be part of the cluster of tissue-related signals.

### pyBASIS — Symphony^TM^ Integration

High-throughput bioinformatic analyses increasingly rely on pipeline frameworks to process data. However, this is often in the context of off-line analyses, i.e. the analyses of data and or/results are decoupled from acquisition. A recent review describes the design philosophies of several current pipeline frameworks, as well as a classification for such frameworks^31^. The Symphony^TM^ pipeline described and utilised in this study would classify as an implicit (syntax)/configuration (paradigm)/commercial (interaction) framework. As mentioned, it can be initiated from the acquisition system in combination with the actual laboratory experiment, or, alternatively, be initiated post-acquisition. The former can arguably be regarded as high-throughput analysis in the true sense due to the seamless integration of both laboratory experiment and bioinformatic interpretation elements.

**Figure 5:**
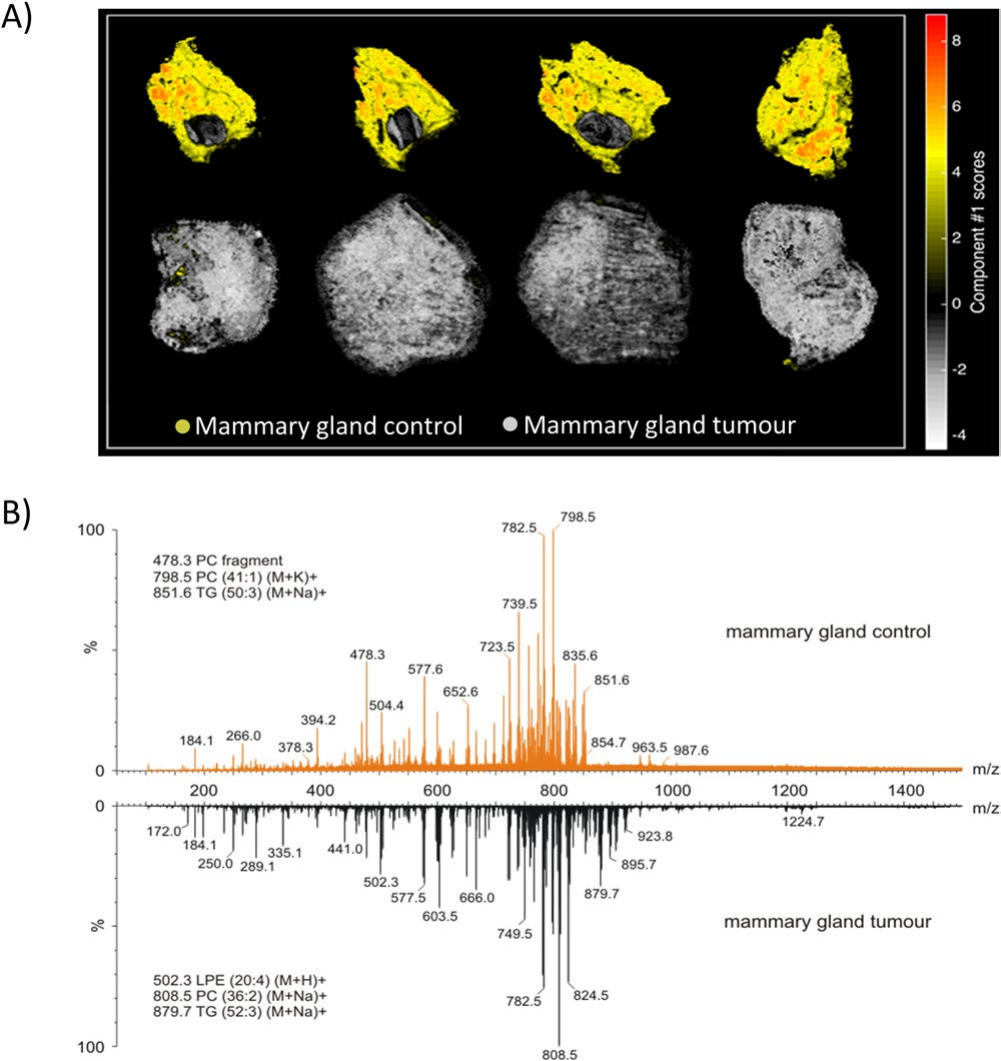
Unsupervised analysis of MALDI-MSI positive ionisation mode imaging datasets, generated on Synapt G2-S*i* Waters mass spectrometer, in breast cancer of mouse models. The first upper row represents 4 control samples taken from healthy animals, where the highlighted regions indicate the healthy tissues, while the lower row indicates solid tumor tissue with minimal (if any) stromal tissue. (**A**) The PCA-driven unsupervised analysis of MALDI-MSI data following the optimized pre-processing strategy separates stromal tissue (yellow/red) from cancerous tissue (white) in mammary breast cancer. (**B**) The representative spectral profiles from mammary gland control and tumour specimens. Shown inset are tentative example identifications.

**Figure 6:**
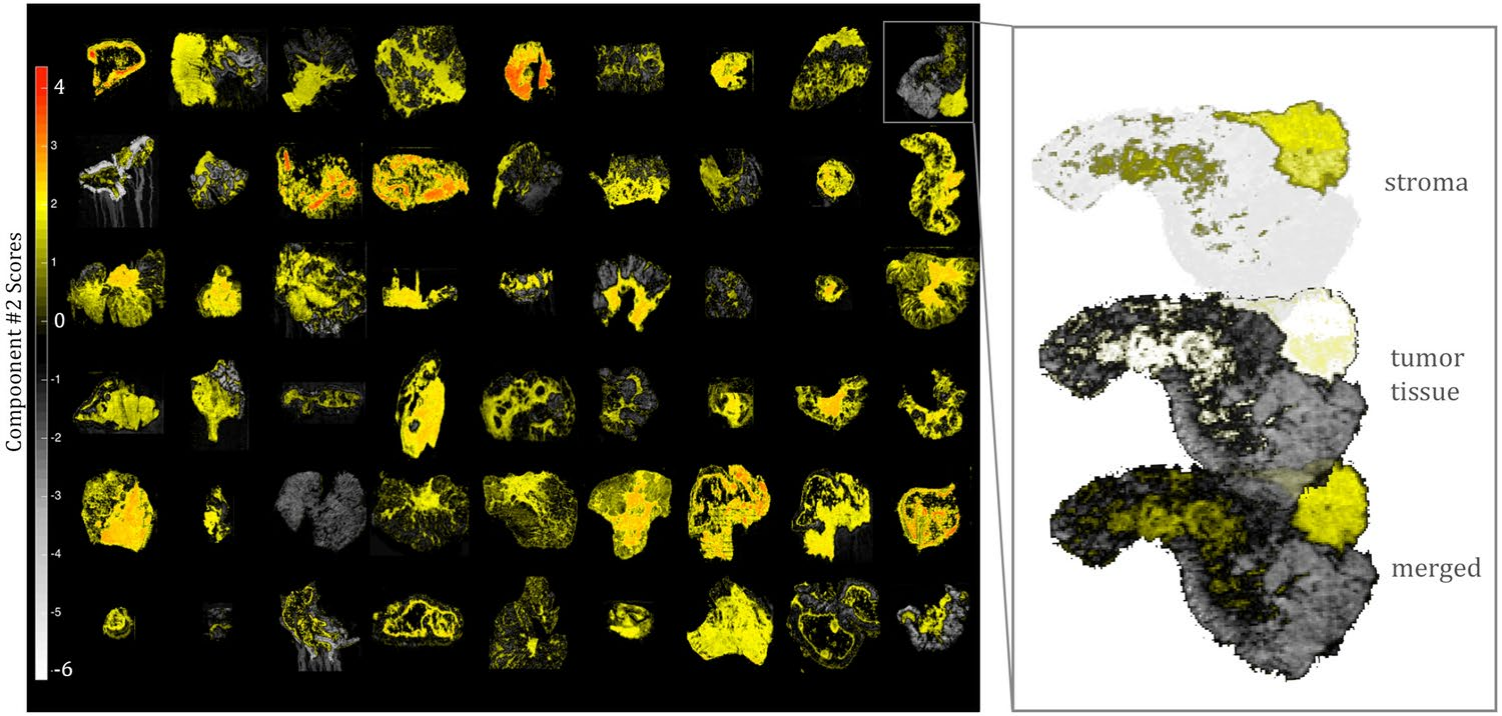
The PCA-driven unsupervised analysis of large-scale DESI-MSI data following the optimized pre-processing strategy separates stromal tissue (yellow) from cancerous tissue (white/grey) in colorectal cancer.

Successful application of the pre-processing steps outlined should ensure that biologically relevant variation is preserved, while features of non-biological origin are minimised. This is demonstrated in Fig. 5 (MALDI-MSI) and Fig. 6 (DESI-MSI), where the largest variation in the pre-processed dataset (captured by the first principal component) is related to separation of molecular ion patterns according to tissue morphology.

## Conclusions

MSI offers an additional layer of spatially resolved chemical information to advance molecular understanding of disease processes and complement current research histopathology. The challenge to translational validation of MSI approaches in clinical research has until now been the lack of capability for scalable and reproducible processing of large scale MSI data. Here, we have introduced a computational open-source platform (pyBASIS) designed to deliver optimized and scalable processing of MSI data for enhanced information recovery and comparative analysis across tissue specimens using machine learning and related pattern recognition approaches. The proposed solution also permits seamless integration of experimental laboratory data and downstream bioinformatics, operating within the Symphony^TM^ environment, resulting in high-throughput translational MSI in the true sense.

## Methods

An integrated bioinformatics solution (pyBASIS) is presented to account for bio-analytical complexities inherent in high-throughput MALDI and DESI-MSI datasets. The informatics pipeline includes a series of modules covering: (a) kernel density based peak alignment to adjust for shifts in m/z measurements across datasets; (b) intra/inter-sample normalization to robustly adjust for scaling differences within and between samples and datasets; (c) cluster-driven removal of matrix/solvent related signals; and (d) variance stabilizing transformation to compensate for intensity-dependent differences in non-biological variance between small and large molecular ion signals. Each module has been designed to operate in an iterative fashion (*i.e*. processing one single sample at a time) and thus is scalable to large MS imaging datasets. The scalable processing is achieved using a high-performance (HDF5) database architecture, in which individual samples are uploaded one at a time into the specific module, processed, deposited back into the database and deleted from memory. This process is iteratively replicated. The output of the pre-processing workflow is a database (‘folder-like’) file (hdf5) containing all processed data (as sub-folders) and associated registered/generated pre-processing parameters (“metadata”) for each workflow step. The database file organization can be easily inspected *via* HDF5Viewer (https://www.hdfgroup.org/downloads/hdfview/). This ensures reproducibility and replicability, offers a mechanism for bioinformatics ‘quality control’, and permits benchmarking of newly acquired data against previously processed data stored within the repository. Here we describe the details of the workflow and demonstrate the pipeline from data collection to the deposition of the pre-processed data, ready for subsequent statistical analyses.

### Mass spectrometry imaging

For all MALDI imaging experiments, a matrix solution of 5 mg/ml α-CHCA dissolved in an acetonitrile/water/TFA (70:30:0.1, v/v/v) solution was deposited onto fresh frozen tissue sections using a SunCollect automated sprayer (SunChrom, Friedrichsdorf, Germany) as described previously^32^. An oversampling technique was applied and the pixel size was fixed to 50 μm. DESI experiments were conducted with a modified 2D linear moving stage (Prosolia, Indianapolis, IN) of which the design, operating conditions and details are described elsewhere^33^. In short, the pixel size was defined as 100 μm, the acquisition rate 5 scans/s, and using methanol/water (95:5, v/v) as the solvent/spray mixture.

MALDI and DESI MSI data analyses were performed using a MALDI Synapt G2-S*i* mass spectrometer (Waters Corporation, Wilmslow, UK) equipped with an Nd:YAG laser operated at 1 kHz. The instrument was calibrated prior to analyses using red phosphorus partially dissolved in acetone and the resolving power was 20,000 FWHM. All analyses were carried out in the positive mode over a mass range of 50–1200 *m/z*. The obtained MSI data were processed, centroided and exported in text file format using High Definition Imaging (HDI) software (Waters Corporation)^33^, which was incorporated in the automation pipeline described below.

Additionally, the previously published MSI dataset of ~100 colon specimens was used for platform validation (see supplementary material). It was acquired using negative ion DESI-MSI analysis, with an Exactive mass spectrometer, Thermo Fisher Scientific coupled with a home-built automated DESI ion source. Spatial resolution for imaging experiments was set to 75 μm. The mass resolution used for all measurements was set to 100,000 with a mass accuracy of <4 ppm. Further details on data acquisition are provided in the previous publication^9^.

### Automation pipeline

To streamline pre-processing steps, pyBASIS components were automated *via* integration within a workflow automation tool called Symphony^TM^ (Waters Corporation, Wilmslow, UK). This is a client/server application that can be triggered by the MassLynx (Waters Corporation) instrument control and acquisition system. In short, a server request is executed, which consists of a list of tasks that are executed upon inputting of data. In this instance, tasks are requests that cover the execution of command line interface driven modules (exe, bat, script). Symphony^TM^ tools provide a means to configure and combine a sequence of tasks, and set up so-called pipeline definition files. The pipeline applied here for MALDI and DESI MSI data processing combines a data transfer step followed by HDI/MALDI chrom (Waters Corporation) peak detection, and pyBASIS MSI data processing. Some of these components are graphically described in Fig. 3.

### Mass to charge (m/z) correction

The pyBASIS package employs a kernel-based “clustering” approach to group and align similar/identical ionic species to a common m/z vector. Using this approach, a histogram is constructed to estimate the frequency of ionic species from sample datasets in distinctive, non-overlapping m/z intervals (“bins”). The bin size of the histogram is a user adjustable parameter (5 ppm by default), and can be defined in absolute (Da) or relative (ppm) units depending on the type of instrument and experimental conditions. The locally estimated scatterplot-smoothing (loess) method is then applied to obtain the smoothed frequency histogram of ionic species over the entire m/z range^34^. This smoothing enhances histogram resolution to 1 ppm for more accurate mass estimation across datasets. Clusters of similar/identical ionic species should appear as peaks on the smooth frequency histogram. The cluster centroids are found when the first derivative of this smooth histogram changes sign, and these are used to denote the common m/z feature vector for all samples. The m/z feature vector of each sample-dataset is then matched to the common one by means of the nearest-neighbour approach^34^. The maximum mass drift is a user adjustable parameter (100 ppm by default) and can be provided in absolute (Da) or in relative (ppm) units.

Additionally, an “internal lock” mass correction algorithm has been implemented for cases where a user provides one or more “reference” ions with known m/z values, ubiquitously distributed in biological tissues^16^. Using this procedure, the m/z drift for each sample dataset is calculated by taking the median of the mass shifts across all matched pairs of experimentally observed and theoretically calculated m/z ratios, within the mass drift window. The m/z of each molecular ion is calibrated by adding or subtracting the estimated mass drift. As with the first approach, the maximum mass drift is a user-adjustable parameter (100 ppm by default) and can be defined in absolute (Da) or in relative (ppm) units.

### Intra-sample normalization

The pyBASIS package implements several *intra*-sample strategies to remove biologically unrelated pixel-to-pixel variation in overall signal intensity^9,29^ by:

1. *“Mean”* (total ion current): Each mass spectrum is normalized to its mean ion current by dividing each peak intensity within a mass spectrum by the mean of all peak intensities of the same spectrum.
2. *“Median”*: Each mass spectrum is normalized to its median ion current by dividing each peak intensity within a mass spectrum by the median of all peak intensities of the same spectrum.
3. *“Median fold change”*: Each mass spectrum is normalized to its median fold change ion current by dividing each peak intensity within a mass spectrum by the median fold change between all peak intensities of the same spectrum and reference spectrum. The reference spectrum is typically chosen to be the median profile across all dataset spectra.

### Inter-sample normalization

The *inter*-sample normalization strategies adjust for sample-to-sample differences in overall signal intensity by dividing all spectra of each sample dataset by a sample-specific scaling factor. The scaling factors are derived based on “homogenized” tissue molecular ion profiles, typically calculated as the average profile across all tissue-related spectra. Similar to intra-sample normalization, the pyBASIS package includes the mean, median and median fold change inter-sample normalization approaches.

1. “*Mean*” (total ion current): Each MSI dataset is normalized to its mean ion current by dividing each peak intensity of a dataset by the mean of all peak intensities of the same dataset.
2. *“Median”* (total ion current): Each MSI dataset is normalized to its median ion current by dividing each peak intensity of a dataset by the median of all peak intensities of the same dataset.
3. *“Median fold change”*: Each MSI dataset is normalized to its median fold change ion current by dividing each peak intensity of a dataset by the median fold change between the peak intensities of the same dataset profile and reference dataset profile. The reference is typically chosen to be the median profile across all samples.

### Variance stabilizing transformation

Here, we assume that the error structure of MS imaging data is characterized by increasing technical variance of molecular level measurements as a function of increased signal intensity, and peak intensities arise through a combination of genuine signals and noise-related signals from different sources, which can be additive or multiplicative in nature^30^. To account for the influence of multiplicative noise, the pyBASIS package employs the started-logarithmic transformation carried out by adding a small constant (“offset”) to the data prior to log transformation. The offset is calculated according to the intensity of the smallest peak^9^. The smallest peak was defined to as 50 counts in our study but can be automatically found by calculating the lower 5% quantile intensity of all peaks above zero.

### Solvent/matrix peak filtering

A cluster-driven peak filtering strategy was employed, which calculates, in an iterative fashion (processing one sample at a time), the correlation matrix of the similarity/difference of signal distribution between m/z features across datasets. The k-means (by default) and Gaussian mixture model algorithms are implemented in the pyBASIS package. Because of its iterative nature, this robust solvent/matrix peak filtering strategy can be applied to large-scale datasets. The workflow currently exports 2 independent clusters of m/z channels across all samples. The tissue related cluster could be easily visually inspected and identified.

### Code availability

The pre-processing Python (py-)BASIS package is freely available under the Apache-2 license at PyPI (link to be provided). The source code, documentation and issue tracking to facilitate the wide use, extensions and validations of the workflow across various use cases are available at https://bitbucket.org/iAnalytica/basis_pyproc.git. The Symphony^TM^ pipelines for the seamless integration of experimental laboratory data with the BASIS pre-processing workflows are also provided within the repository.

## Electronic supplementary material

Supplementary Information
