# Supplementary material for: BASIS: High-performance bioinformatics platform for processing of large-scale mass spectrometry imaging data in chemically augmented histology
Source: Sci Rep. 2018 Mar 6;8:4053. doi: 10.1038/s41598-018-22499-z (PMC5840264; doi:10.1038/s41598-018-22499-z)
Supplement: Supplementary file 1 — Supplementary Information [file 41598_2018_22499_MOESM1_ESM.pdf]

BASIS: High-performance bioinformatics platform for processing of large-scale mass spectrometry imaging data in chemically augmented histology

Kirill Veselkov[1,*]; Jonathan Sleeman[2,3]; Emmanuelle Claude[4]; Johannes PC Vissers[4]; Dieter Galea[1]; Anna Mroz[1], Ivan Laponogov[1]; Mark Towers[4]; Robert Tonge[4]; Reza Mirnezami[1]; Zoltan Takats[1]; Jeremy K Nicholson[1], James I Langridge[4]

[1] Computational and Systems Medicine, Department of Surgery and Cancer, Imperial College London, UK, [2] University of Heidelberg, Medical Faculty Mannheim, Center for Biomedicine and Medical Technology, Mannheim, Germany; [3] KIT Karlsruhe, Institute of Toxicology and Genetics, Karlsruhe, Germany; [4] Waters Corporation, Wilmslow, UK. [*]To whom correspondence should be addressed. Email: kirill.veselkov04@imperial.ac.uk

SUPPLEMENTARY INFORMATION

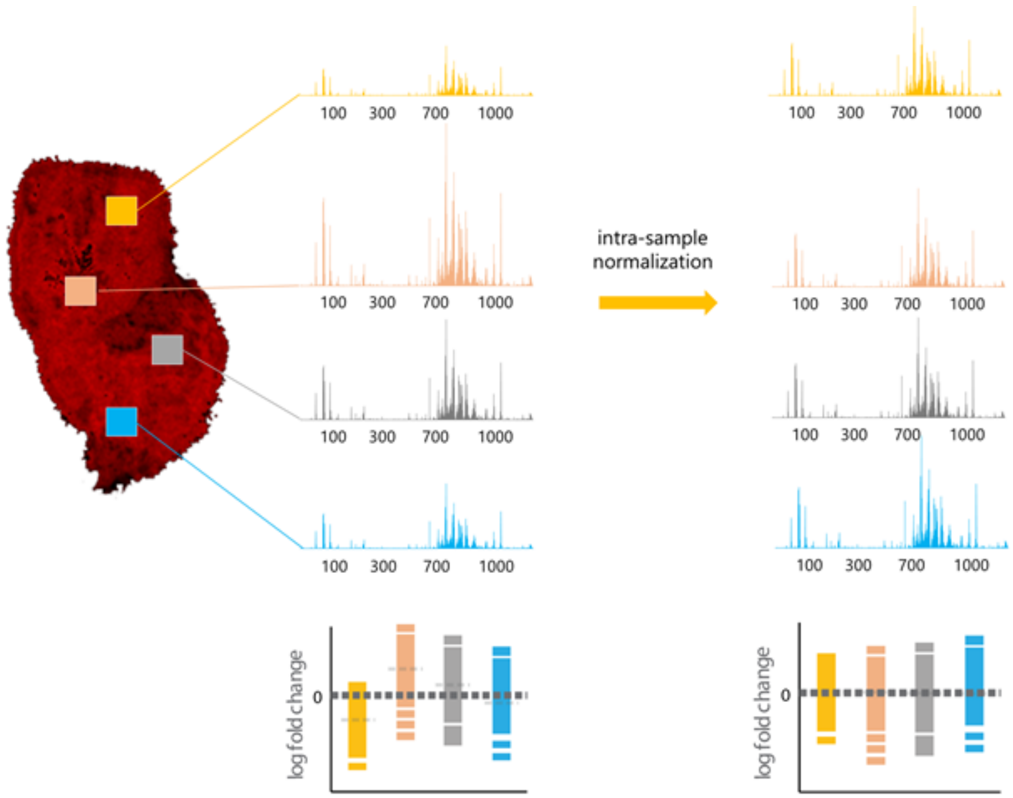

Figure S1. Intra-sample normalization strategy. Spectra with different median log fold changes are zero-centred in order to account for the difference in total ion intensities between spectra within the same sample (intra-sample normalization).

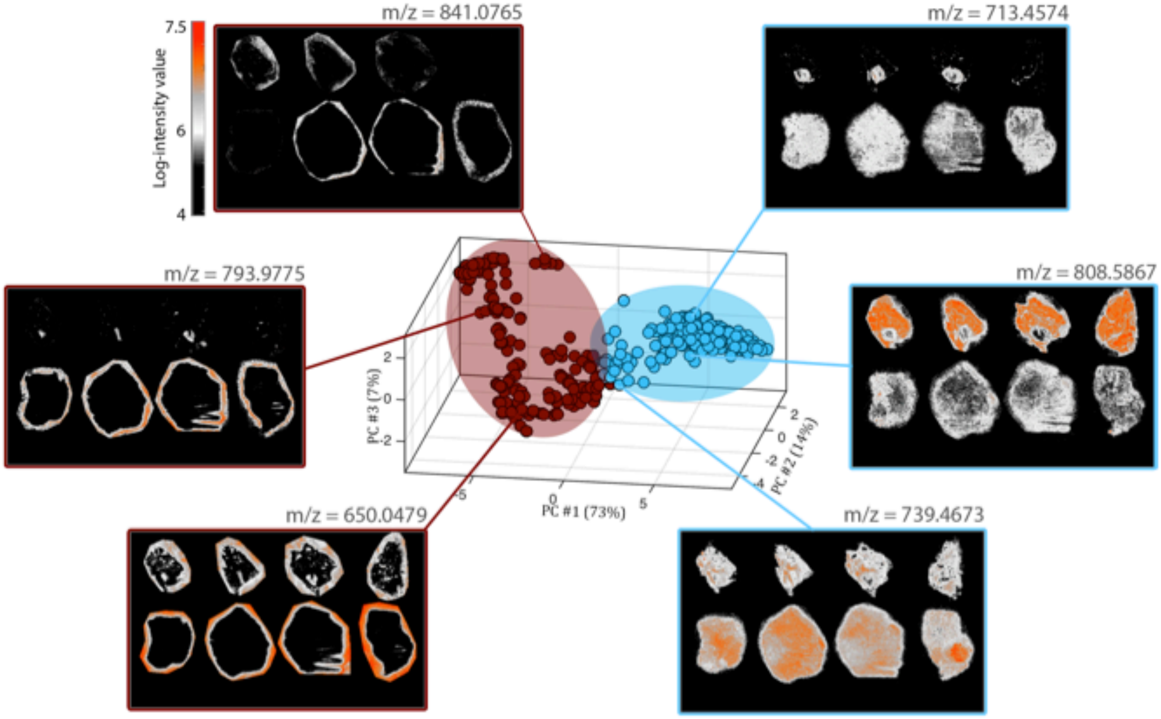

Figure S2. Cluster-driven matrix/solvent removal strategy for large-scale MSI data. The negative intensity correlation between tissue (matrix) and solvent is utilized to perform clustering and subsequent separation of tissue- and background-related signals. The differentiability of such clusters is also visualized by a PCA plot.
